# Successful Strategies to Engage Research Partners for Translating Evidence into Action in Community Health: A Critical Review

**DOI:** 10.1155/2015/191856

**Published:** 2015-03-01

**Authors:** Jon Salsberg, David Parry, Pierre Pluye, Soultana Macridis, Carol P. Herbert, Ann C. Macaulay

**Affiliations:** ^1^Participatory Research at McGill, Department of Family Medicine, McGill University, 5858 Chemin de la Côte-des-Neiges, Suite 300, Montreal, QC, Canada H3S 1Z1; ^2^Department of Family Medicine, McGill University, 5858 Chemin de la Côte-des-Neiges, Suite 300, Montreal, QC, Canada H3S 1Z1; ^3^Department of Kinesiology & Physical Education, McGill University, 475 Pine Avenue West, Montreal, QC, Canada H2W 1S4; ^4^Department of Family Medicine, Schulich School of Medicine & Dentistry, University of Western Ontario, 1151 Richmond Street, London, ON, Canada N6A 3K6

## Abstract

*Objectives*. To undertake a critical review describing key strategies supporting development of participatory research (PR) teams to engage partners for creation and translation of action-oriented knowledge. *Methods*. Sources are four leading PR practitioners identified via bibliometric analysis. Authors' publications were identified in January 1995–October 2009 in PubMed, Embase, ISI Web of Science and CAB databases, and books. Works were limited to those with a process description describing a research project and practitioners were first, second, third, or last author. *Results*. Adapting and applying the “*Reliability Tested Guidelines for Assessing Participatory Research Projects*” to retained records identified five key strategies: developing advisory committees of researchers and intended research users; developing research agreements; using formal and informal group facilitation techniques; hiring co-researchers/partners from community; and ensuring frequent communication. Other less frequently mentioned strategies were also identified. *Conclusion*. This review is the first time these guidelines were used to identify key strategies supporting PR projects. They proved effective at identifying and evaluating engagement strategies as reported by completed research projects. Adapting these guidelines identified gaps where the tool was unable to assess fundamental PR elements of power dynamics, equity of resources, and member turnover. Our resulting template serves as a new tool to measure partnerships.

## 1. Introduction

The creation and timely translation of action-oriented knowledge can rest on meaningful engagement with end-users, even before the research begins [1, 2]. Participatory research (PR) (following Cargo and Mercer [3] and Green et al. [4] and we use PR as an umbrella term to include all partnered research, including community-based participatory research (CBPR), action research, participatory action research, participatory evaluation, community engagement and patient engagement), and community engagement continue to attract increased attention as an approach to research, requiring formation of teams of researchers in partnerships with those affected by the issue under study in the community [3–5] and those who will utilize the results to effect change [6, 7]. Overall, the literature suggests that the PR partnership approach increases the relevance of research questions [3, 5, 8], with the potential for effective knowledge translation [9, 10], leading to faster uptake of evidence into practice [11]. For these reasons research granting agencies, including the National Institutes of Health (NIH), the Patient Centered Outcomes Research Institute (PCORI), and the Canadian Institutes of Health Research (CIHR), are increasingly requiring that researchers partner with community members, patients, health professionals, health organisations, and policy makers, resulting in many more researchers adopting a participatory approach.

In 1995, Green and colleagues developed guidelines intended to allow reviewers of funding agencies to assess stakeholders' engagement in PR projects [4, 12]. In 2008, these guidelines were further refined and reliability was tested to develop the* Reliability Tested Guidelines for Assessing Participatory Research Projects* [13] as a tool to (i) help funding agencies and peer reviewers to assess the participatory nature of proposals submitted for funding as participatory research; (ii) aid evaluators in assessing the extent to which projects meet participatory research criteria; and (iii) assist researchers and intended users of the research (i.e., nonacademic partners) in strengthening the participatory nature of their project proposals and applications for funding [12, 13]. In 2009 van Olphen et al. [14] applied these guidelines for the first time, to a single project to assess to what extent their research was participatory as perceived by community, advocacy, and scientific partners. The authors concluded that this had been a very useful undertaking and that “further research should focus on the adaptation of PR principles to assist in evaluating the process and outcomes of PR [14].”

As the principles of the PR approach are used in a wide variety of research and contexts, there is a need to explore the following questions: What are the key processes of PR and what are the practical ways to achieve equitable partnerships? What processes support the constant negotiation between all team members for research goals and objectives, partner roles and responsibilities, and decision-making procedures, together with balancing knowledge generation with the need for action? Therefore, the purpose of this study is to build on recommendations [14] and use the 2008* Reliability Tested Guidelines* to undertake a critical literature review of PR projects to synthesize key practical strategies that foster a successful PR process, resulting in continuous discussions between partners that will in turn facilitate knowledge translation activities throughout the research [15].

## 2. Materials and Methods

### 2.1. Data Sources

A critical review goes beyond the description of primary studies and includes an empirical analysis for exploring new ideas [16]. While critical reviews are criticized for their nonsystematic approach, “the ‘critical' component of this type of review is* key* to its value” [16].

**Figure 1:**
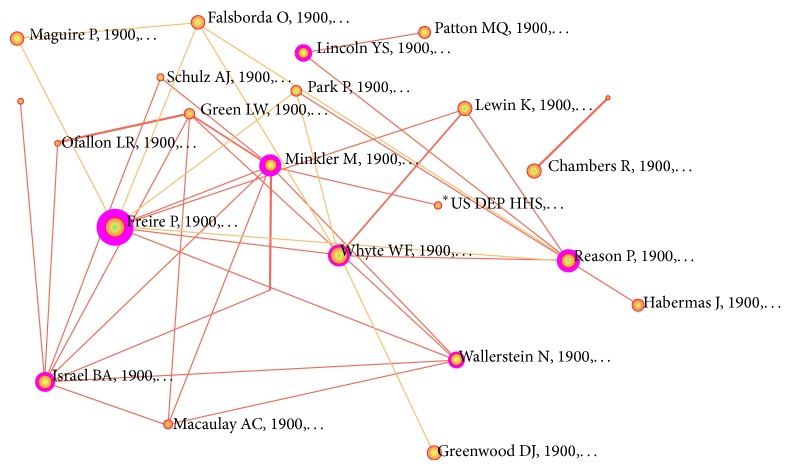


To begin, a multidisciplinary bibliographic database (ISI Web of Science) was searched using the phrase “participatory research” for all articles from 1995 (when the initial PR guidelines were published) until October 2009 (which was the year after the* Reliability Tested Guidelines *were published). Results of this search yielded 1866 publications. These were then imported into CiteSpace—a bibliometric network analysis tool (http://cluster.cis.drexel.edu/~cchen/citespace/)—which generated a map of author-citation frequency. Results contained foundational PR scholars such as Paulo Freire and theoreticians including Peter Reason as well as those with practical PR experience. Our selection tool eliminated theoretical/foundational authors and retained only authors that have conducted practical PR studies. For this review we needed to limit the size of the study and chose to retain only the top four leading PR practitioners using their CiteSpace centrality scores: Barbara A. Israel, Meredith Minkler, Nina Wallerstein, and Ann C. Macaulay (Figure 1).

Next, a librarian-mediated search was conducted for all published materials by these four authors in PubMed, Embase, ISI Web of Science, PsychInfo, and CAB (Ovid database) for abstracts between January 1995 and October 2009. In addition we also reviewed chapters from books edited by these authors [17–19]. Duplicates were removed, for a total of 151 records (title, authors, source, and abstract).

### 2.2. Study Selection

**Figure 2:**
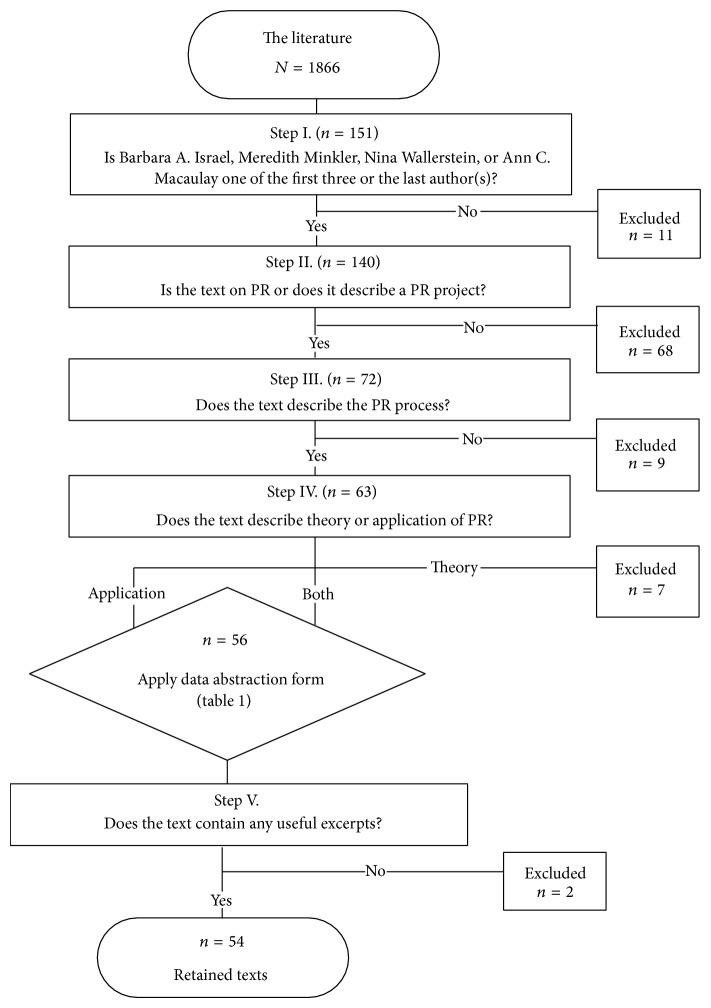


A staged selection process was then completed to limit the sample using eligibility criteria. First, records were excluded when one of the abovementioned PR leaders was neither one of the first three authors nor the last author (*n* = 11) to ensure that the leader had substantive input into the work. The second step excluded records that were not PR related (*n* = 68). The third step excluded records that did not contain any description of the PR process (*n* = 9) or records that contained only the theory of PR (*n* = 7). In the final step, records were excluded when they did not contain useful excerpts (*n* = 2), leaving 54 retained records (Figure 2).

### 2.3. Data Extraction

We conducted a deductive qualitative thematic analysis to extract useful data from our sample of documents [20]. For each of the 54 retained documents, relevant excerpts were selected and compiled in a Word document and organized by theme. These themes were derived from the partnership-related dimensions of the* Reliability Tested Guidelines for Assessing Participatory Research Projects* [13]. These guidelines contain 25 questions, 21 of which target the PR partnership process, making them very suitable to serve as themes for data extraction and analysis. These questions informed our coding scheme to identify PR process strategies. Using a coding grid based upon these questions (Table 1), partnership process-specific excerpts from the retained documents were extracted for analysis. Each retained document was reviewed in its entirety, and all excerpts in those documents that directly answered one of the questions were extracted and compiled in a matrix of “data by theme” for further analysis. Data coding was nonexclusive, and each excerpt could be coded to one or more questions on the coding grid.

### 2.4. Data Analysis

Data abstraction and coding were undertaken by one author (David Parry) using nonspecialized software (MS Word), which is appropriate for a deductive qualitative data analysis using a limited number of themes (codes). Each excerpt extracted from the retained documents was assigned to one or more themes, which was verified by a second author (Pierre Pluye or Jon Salsberg). Disagreements were discussed for possible resolution, and any that could not be resolved were adjudicated by a third party (Jon Salsberg for Pierre Pluye and vice versa). Using a constant comparative technique, themes were collapsed into overarching categories. These categories were generated through initial and focused coding techniques by comparing and contrasting text segments and sorting codes into conceptually meaningful units [21]. For example, subthemes such as “advisory committee,” “steering committee,” and “planning committee” were all grouped under the main theme “committee.”

## 3. Results 

Table 2 presents the references of the 54 documents that were retained for analysis and are organized by the four main authors. From these documents, 186 excerpts were assigned or coded to one or more than one theme. Of those, there was agreement between the reviewers for 180 (97%) of excerpts. For the six remaining excerpts where there was disagreement, consensus was reached on five, and final judgment was sought from a third author (Jon Salsberg) for one excerpt.

The five most frequently mentioned strategies for fostering a researcher-community partnership are listed (unranked) and described in Table 3. These are forming an advisory board, developing a research agreement, using group facilitation techniques, hiring from the community, and having frequent meetings.

The remaining less frequently mentioned strategies are summarized in Table 4, which we felt could not be collapsed into categories without losing individual substance. However we consider these examples as also being extremely important for researchers to put into practice, including the need for researchers to make active efforts to reach out and learn about their partners and their communities; facilitating engagement by being flexible and working around schedules of the partners; understanding community priorities and culture; establishing clear lines of communication; speaking frankly and agreeing to disagree; building community capacity; supporting partners interpretation of data; publishing results in community; including nonacademic partners as copresenters and coauthors; working with community partners to build resources based on results; using the results to influence policy; and regular evaluation of the partnerships.

## 4. Discussion

This is a first step in a larger research agenda to identify variation in PR practices across contexts and partnership stages that could in the future be drawn on to answer the question of efficacy of PR practices. As this review was exploratory and not systematic, we decided to include a purposeful sample of included studies. CiteSpace helped us to elicit a criterion for a purposeful sampling. The rationale was that most cited papers for our review played a role similar to “key informants” in primary research. Given that this study had limited resources, we focused on the top four authors (most popular “key information resources”).

From the four authors identified, committees such as steering committees and advisory committees are the most frequently mentioned strategy as a way to engage key stakeholders around the table from the beginning—including patients, practitioners, service managers, communities and the public, and policy makers. The second most frequently mentioned strategy is drafting research agreements, which some recommend should be done early in the partnership in order to avoid misunderstandings and because the process of developing written agreements or partnership principles is in itself a partnership building process [72, 74]. However, the authors of this paper are also aware of teams who have not wanted a written agreement, either for cultural reasons where a verbal agreement is deemed very final, or due to the fact that it could be construed to imply lack of trust between the researchers and the partners. Our review results show that group facilitation is often suggested as a way to offer equal opportunity for partners to participate in discussions and to afford more reserved partners the chance to voice their opinion. Facilitation includes informal group discussions and formal techniques with many techniques borrowed from management. Hiring staff from the community increases credibility of the research, adds cultural relevance, builds capacity, promotes empowerment, provides work, brings in finances, and integrates knowledge translation throughout the process. Finally, frequent meetings are essential to maintain open communication as research evolves and to manage different expectations.

Table 4 shows many other additional practical strategies and supports the importance of meeting the needs of various partnerships in a wide range of contexts. It also emphasises the need for researchers to learn more about community issues and fully engage community members throughout the research process including interpretation of data and dissemination of results both internally within the community where the research was undertaken and externally.

To our knowledge, this is the first time the* Reliability Tested Guidelines* have been used to undertake a critical literature review to document PR partnership processes. The strengths of this review include (i) using a bibliometric methodology to identify leading PR practitioners, (ii) a comprehensive identification of PR studies conducted by these authors, (iii) a transparent selection of relevant documents describing PR partnership processes, and (iv) a reproducible deductive qualitative data thematic analysis using the* Reliability Tested Guidelines* as basis for a coding scheme to analyze relevant excerpts from retained documents. This critical review has also identified that the four authors reviewed utilise these processes and also reestablished the* Reliability Tested Guidelines* as reliable criteria by which to measure partnerships.

It is noteworthy that four or fewer excerpts were identified for the following* Reliability Tested Guidelines* dimensions: mutual learning (Q8), conflict resolution over interpretation of results (Q14), and data ownership and sharing (Q15). This is surprising considering that mutual learning is a fundamental PR principle and the latter two are key issues to be resolved for any PR project. More literature on these topics would be very useful; for example, Jagosh and colleagues found that successful conflict resolution led to further strengthening of the teams [75].

This review also highlights gaps that the* Reliability Tested Guidelines* do not address. These include (i) the issues of power dynamics and recommendations for ways of decentralizing power and decision-making either through subcommittees or through a high level of local control, (ii) ways to address issues of equity of resources, that is, equitable sharing of resources across community organizations and researchers, or providing grants or other funding to participating community-based organizations, and (iii) the common problem of adding or replacing new members throughout the project—which causes shifting group dynamics. We also recognize that more other human aspects of partnerships have not been addressed, including the time needed to consolidate partnerships, issues of power differences, personality clashes, and institutional cultures.

There is much diversity in the strategies discussed by the four PR leaders. This is particularly encouraging for three reasons. First, it suggests that PR is highly adaptable to many contexts and settings and the iterative nature of this research approach. As PR is rapidly expanding beyond its earlier application in health promotion with marginalized communities, this adaptability will become increasingly important for partnerships with new types of communities including communities of practice and organizations such as practice-based research networks [76] and also for partnering with patients and policy makers. Second, research teams can find many strategies in the results to draw upon when starting out. A given strategy does not always work for a given context and the whole team can discuss potential alternative strategies. Third, the diversity of results reinforces the notion that the PR process is an active, iterative endeavour, requiring energy and flexibility from all partners. The findings are supported by other authors including a critical review by Cargo and Mercer [3] and incorporated by Wallerstein and Duran [10] in a conceptual logic model of community-based participatory research. For those embarking on PR there are recommendations and training curricula from individual teams [76–78] and organisations [74, 79–81] on how to build PR teams and maintain equitable partnerships throughout the research process, including dissemination of the results. There are also an increasing number of publications on the experiences of both academic [82] and community [83, 84] team members from their participatory research experiences and documented common characteristics of successful community-institutional partnerships [85].

While this review provided an innovative synthesis of key PR strategies for researchers using a PR approach, a limitation is that it is based on only four authors' publications. Because the review included book chapters not limited to the word count restrictions of journal articles we may have captured more details than from journal articles alone. There are no standard recommendations for reporting on PR; from this review we recommend that journal editors require the key stages from the* Reliability Tested Guidelines* to be included, which would facilitate future synthesis. Our results consist of strategies that could be tested and explored in greater detail through a larger systematic literature review, which may include more detailed descriptions of applied strategies for planning and sustaining PR partnerships. Such a systematic review might be able to rank these strategies in terms of their effectiveness in different contexts, which would first require further basic research into the efficacy of particular participatory strategies and their effectiveness in generating and translating new knowledge into action. As PR is becoming more accepted, this new evidence is slowly emerging within the fields of participatory research as well as in implementation and translational science.

## 5. Conclusion

This review is the first to adapt the* Reliability Tested Guidelines for Assessing Participatory Research Projects* to identify leading processes that support PR partnerships. Five key practical strategies to foster a successful PR process are identified that in turn integrate knowledge translation throughout the research process. Some of these results have already been incorporated into the Canadian Institutes for Health Research (CIHR)* Guide to Researcher and Knowledge-User Collaboration in Health Research *[81]. One colleague remarked, “I will print these 5 strategies in big color letters and pin them in front of my desk. No one can remember 25 questions, while anybody can handle 5 ideas per day.” The guidelines, originally intended to allow funders to assess partnership engagement in grant applications, proved effective at identifying and evaluating the same engagement strategies as reported by completed research projects. Adapting these guidelines for our use identified gaps where the tool was unable to assess the fundamental PR elements of power dynamics, equity of resources, and member turnover. Our resulting template serves as a new tool for research teams to apply to measure their own partnerships.

## Figures and Tables

**(a):** 

Number	Question	Mercer	Rationale
Q1	How are the needs of the project's participants considered?	1(b) Is the mix of participants included in the research process sufficient to consider the needs of the project's intended users?	Participants' needs. Although this question asks more about the composition of the participants, it can be construed to enquire about how the needs of the intended users are considered.

Q2	How are the barriers to participation in the research project by the intended users addressed, especially those who might otherwise be under-represented?	1(c) Is effort made to address barriers to participation in the research process by intended users who might otherwise be under-represented?	Barriers to participation.

Q3	How has trust between the researchers and intended users been established?	1(d) Has provision been made to build trust between researchers and intended users participating in the research process?	Trust.

Q4	How have researchers and intended users decided to jointly manage the project?	1(e) Do the researchers and intended users participating in the research process have an explicit agreement (verbal or written) regarding management of the project?	Project management. Although the question is about an explicit agreement, the main theme is project management. This is a key question for PR process. *N.B.* This question was modified half way into data collection. Decision to modify was mitigated by the fact that we had already captured whether written agreements existed or not.

Q5	How was (were) the research question(s) collaboratively developed between researchers and intended users?	2(a) Was (were) the research question(s) developed through a collaborative process between researchers and intended users?	Collaborative development of research question.

Q6	How does the research plan to build the capacity of the intended users to address broader determinants of health?	2(e) Does the proposed research project plan to build the capacity of intended users to address individual and/or broader determinants of health?	Capacity building. *N.B.* Green uses empowerment, which was originally used.

Q7	How does the research process apply the knowledge of intended users in the phases of (1) conceptualizing/designing, (2) planning, (3) implementation, (4) data collection, and (5) evaluation?	2(b) Has the proposed research project applied the knowledge and experience of intended users in conceptualizing and/or designing the research? 3(a) Does the proposed research project apply the knowledge and experience of intended users in the implementation of the research? 3(e) Does the proposed research project provide intended users with opportunity to participate in planning and/or executing the data collection (whether or not the intended users choose to take that opportunity)?	Involvement in all research phases. Merely combined Mercer's questions for simplicity.

Q8	How does the research project provide for mutual learning between intended users and researchers?	2(c) Does the proposed research project provide for the mutual learning among intended users and researchers?	Mutual learning.

Q9	How does the research process allow for the intended users to learn about research methods?	3(b) Does the proposed research project provide intended users participating in the research process with opportunity to learn about research (whether or not the intended users choose to take that opportunity)?	Intended users learning about research methods. Reference to “opportunity” is removed because it is irrelevant here as it is not being applied as an evaluation tool

Q10	How does the research process allow for the researchers to learn about the user perspective on the issue(s) being studied?	3(c) Does the proposed research project provide researchers with opportunity to learn about user perspectives on the issue(s) being studied?	Researcher learning about user perspective.

Q11	How does the research process allow for mutual decision-making in changing research methods or focus?	3(d) Do the researchers and intended users participating in the research process have an explicit agreement (verbal or written) regarding mutual decision-making about potential changes in research methods or focus?	Mutual decision-making. Reference to explicit agreement is removed as it is not an important aspect of question, and we had already captured whether there was a written agreement or not.

Q12	How are the intended users involved in analysis and interpretation?	3(f) Does the proposed research project provide intended users with opportunity to participate in planning and/or executing the analysis (whether or not the intended users choose to take that opportunity)? 3(g) Are plans to involve intended users in interpreting the research findings sufficient to reflect the knowledge of the particular context and circumstances in the interpretation?	Merged questions for simplicity. *N.B.* It was modified to reflect Mercer's working half way through data collection.

Q13	How does the research process reflect a commitment by researchers and intended users to social, individual or cultural actions consequent to the learning acquired through research?	4(a) Does the proposed research project reflect sufficient commitment by researchers and intended users participating in the research process to action (e.g., social, individual, and/or cultural) following the (learning acquired through) research?	Action based upon research results. Wording simplified (removed brackets).

Q14	How is the process set out for acknowledging and resolving differences between researchers and intended users over interpretation of research results?	4(b) Do the researchers and intended users engaged in the research process have an explicit agreement (verbal or written) for acknowledging and resolving in a fair and open way any differences in the interpretation of research results?	Conflict resolution. Reference to explicit agreement is removed as it is irrelevant, and we had already captured whether there was a written agreement or not.

Q15	How is the process set out regarding the issue of data ownership and sharing?	4(c) Do the researchers and intended users engaged in the research process have an explicit agreement (verbal or written) regarding ownership and sharing of the research data?	Data ownership and sharing. Reference to explicit agreement is removed as it is irrelevant, and already recording whether written agreement or not.

Q16	How do the researchers and intended users jointly disseminate research results?	4(e) Do the researchers and intended users engaged in the research process have an explicit agreement (verbal or written) regarding the dissemination (and/or translation or transfer) of research findings? 4(f) Does the proposed research project provide intended users with opportunity to participate in dissemination of project findings to other intended users and researchers (whether or not the intended users choose to take that opportunity)?	Dissemination. Questions combined and simplified.

Q17	How is feedback of research results to intended users handled?	4(d) Do the researchers and intended users engaged in the research process have an explicit agreement (verbal or written) regarding feedback of research results to intended users?	Feedback of research results.

**(b):** 

Excluded questions		
Number	Question	Rationale for exclusion
Mercer 1(a)	Are the intended users (may include users, beneficiaries, and/or stakeholders) of the research described in a way sufficient to assess their representation in the project?	Question pertains to the research proposal itself and not the project.

Mercer 1(b)	Is the mix of participants included in the research process sufficient to consider the needs of the project's intended users?	Question examines the composition of the community partners and not the comanagement structures of the researcher-partner interface.

Mercer 2(d)	Does the proposed research project consider multiple levels of determinants of health (e.g., individual, familial, organizational, political, and/or economic)?	Question examines the nature and content of the research itself and not the comanagement structures that exist between the researchers and intended users.

Mercer 4(g)	Is there sufficient provision for assistance to intended users to indicate a high probability of research results being applied?	Question concerns the outcomes and uptake of the research and not the process itself.

**Table 2:** Summary of retained records for review organized by author.

Author(s)	Year	Title
Barbara A. Israel		
[22]	1995	Redesigning Work Systems to Reduce Stress: A Participatory Action Research Approach to Creating Change
[23]	1997	“It's a 24-Hour Thing … A Living-for-Each-Other Concept”: Identity, Networks, and Community in an Urban Village Health Worker Project
[5]	1998	Review of Community-Based Research: Assessing Partnership Approaches to Improve Public Health
[24]	1996	Role of Control and Support in Occupational Stress: An Integrated Model
[25]	1998	Detroit's East Side Village Health Worker Partnership: Community-Based Lay Health Advisor Intervention in an Urban Area
[26]	1998	Conducting a Participatory Community-Based Survey for a Community Health Intervention on Detroit's East Side
[27]	1999	Establishing LA VIDA: A Community-Based Partnership to Prevent Intimate Violence against Latina Women
[28]	2001	The Detroit Community-Academic Urban Research Center: Development, Implementation, and Evaluation
[29]	2001	Can Communities and Academia Work Together on Public Health Research? Evaluation Results from a Community-Based Participatory Research Partnership in Detroit.
[30]	2001	The East Side Village Health Worker Partnership: Integrating Research with Action to Reduce Health Disparities
[31]	2002	The Relationship between Social Support, Stress, and Health among Women on Detroit's East Side.
[32]	2002	Addressing Social Determinants of Health through Community-Based Participatory Research: The East Side Village Health Worker Partnership
[33]	2003	Commentary: Model of Community Health Governance: Applicability to Community-Based Participatory Research Partnerships
[34]	2003	Community Action against Asthma: Examining the Partnership Process of a Community-Based Participatory Research Project
[35]	2003	Engaging Women in Community Based Participatory Research for Health
[36]	2003	Evaluation of a Partnership Approach to Translating Research on Breast Cancer and the Environment
[37]	2004	Identification of Gaps in the Diagnosis and Treatment of Childhood Asthma Using a Community-Based Participatory Research Approach
[38]	2004	Application of Health Promotion Theories and Models for Environmental Health
[39]	2004	Assessing and Strengthening Characteristics of Effective Groups in Community-Based Participatory Research Partnerships
[40]	2005	Strategies and Techniques in Effective Group Process in CBPR Partnerships
[41]	2005	Community Involvement in the Conduct of a Health Education Intervention and Research Project: Community Action against Asthma
[42]	2005	Documentation and Evaluation of CBPR Partnerships
[43]	2005	Community-Based Participatory Research: Lessons Learned from the Centers for Children's Environmental Health and Disease Prevention Research
[44]	2005	Developing and Implementing Guidelines for Dissemination: The Experience of the Community Action against Asthma Project
[45]	2006	Challenges and Facilitating Factors in Sustaining Community-Based Participatory Research Partnerships: Lessons Learned from the Detroit, New York City and Seattle Urban Research Centers
[46]	2006	Engaging Urban Residents in Assessing Neighborhood Environments and Their Implications for Health
[47]	2008	Evaluation of Community Action against Asthma: A Community Health Worker Intervention to Improve Children's Asthma-Related Health by Reducing Household Environmental Triggers for Asthma

Meredith Minkler		
[48]	1995	Combining Research, Advocacy, and Education: The Methods of the Grandparent Caregiver Study
[49]	1996	Health of Grandmothers Raising Children of the Crack Cocaine Epidemic
[50]	1999	Grandparents as Parents: A Survival Guide for Raising a Second Family
[51]	2000	Using Participatory Action Research to Build Healthy Communities
[52]	2001	Contributions of Community Involvement to Organizational-Level Empowerment: The Federal Healthy Start Experience
[53]	2003	Ethical Challenges in Community-Based Participatory Research
[54]	2003	Ethical Challenges in Community Based Participatory Research: A Case Study from the San Francisco Bay Area Disability Community
[55]	2003	Attitudes of People with Disabilities toward Physician-Assisted Suicide Legislation: Broadening The Dialogue
[56]	2003	Community-Driven Asset Identification and Issue Selection
[57]	2003	Influencing Policy through Community-Based Participatory Research
[58]	2004	Ethical Challenges for the “Outside” Researcher in Community-Based Participatory Research
[59]	2005	Community-Based Research Partnerships: Challenges and Opportunities
[60]	2008	Promoting Environmental Justice through Community-Based Participatory Research: The Role of Community and Partnership Capacity
[61]	2006	Sowing the Seeds for Sustainable Change: A Community-Based Participatory Research Partnership for Health Promotion in Indiana, USA and Its Aftermath
[62]	2006	Promoting Environmental Health Policy through Community Based Participatory Research: A Case Study from Harlem, New York

Nina Wallerstein		
[63]	1999	Power between Evaluator and Community: Research Relationships within New Mexico's Healthier Communities
[64]	2000	Community-Based Prevention: Programs That Work
[65]	2003	The Dance of Race and Privilege in Community-Based Participatory Research
[66]	2004	Intermediate Outcomes of a Tribal Community Public Health Infrastructure Assessment
[67]	2004	Bridging Community Intervention and Mental Health Services Research
[68]	2005	Developing and Maintaining Partnerships with Communities
[69]	2006	Woman to Woman: Coming Together for Positive Change-Using Empowerment and Popular Education to Prevent HIV in Women
[70]	2006	Commentary: Challenges for the Field in Overcoming Disparities through a CBPR Approach
[71]	2006	Using Community-Based Participatory Research to Address Health Disparities

Ann C. Macaulay		
[72]	1998	Participatory Research with Native Community of Kahnawake Creates Innovative Code of Research Ethics
[73]	1999	Participatory Research Maximises Community and Lay Involvement

**Table 3:** Summary and description of the most frequently mentioned strategies for developing a research-community partnership.

Strategy	Description
(1) Development of an advisory committee	(i) Acomposition of researchers, the intended users of the research, and/or representatives of community organizations (ii) Advisory committees allow for inclusion of all viewpoints throughout the research process and joint development of dissemination strategies and action plans (iii) Subcommittees are often used to divide up tasks (e.g., reviewing new proposed research topics, articles for publication, partnership evaluation)

(2) Development of research agreements	(i) Before the research begins, clearly spell out researchers and partner roles and responsibilities, outline how decisions will be made (e.g., by consensus or by voting), and set out what to do if conflict arises (ii) Research agreements may also include plans for data ownership and control, interpretation of data, and procedures for resolving disagreement over research results (iii) Developing agreements is seen as a trust-building exercise

(3) Use of group facilitation techniques	(i) Can be both a formal and an informal process to ensure meaningful involvement and participation of partners (ii) Formal facilitation includes focus groups, workshops, and nominal group techniques (iii) Informal techniques include circulating agendas ahead of time, small group work, and one-on-one informal discussions

(4) Hiring staff from the community of study	(i) Hiring local persons as project staff recognizes community members' abilities to establish good relationships with individual participants for recruitment and ongoing data collection (ii) Projects hire well-respected community members as a “community champions,” field coordinators, intervention staff, interviewers, and group cofacilitators, for data collection and analysis.

(5) Frequent communication	(i) Communication between partners through regular group meetings to keep all partners updated on progress and changes in procedures and as a way of discussing concerns and challenges (ii) Other methods include telephone calls to partners who missed meetings to bring them up-to-date and prompt circulation of meeting minutes and newsletters

**Table 4:** Summary and description of less frequently mentioned strategies for developing a research-community partnership.

Strategy	Examples
(i) Researchers need to make active efforts to learn about the participants and their context	(i) Attending community-organized educational sessions or going on a community tour (ii) Arranging retreats with community members (iii) Organizing structured workshops with community members, as well as having informal conversations with them (iv) Conducting formal interviews with community organizations (v) Actively involving intended users through hiring study staff from the community and utilizing a community organizer/champion (vi) Forming advisory board for the project with representation from organizations implicated in the research

(ii) Facilitate intended user involvement	(i) Be flexible with partners' work schedules and negotiate with their employers for study-related tasks (ii) Utilize community contacts for recruitment of marginal community members or make use of “snowball” referral (iii) Reach out to places frequented by community members (e.g., schools) (iv) Adopt group facilitation techniques (v) Approach partners individually for input away from larger groups (vi) Understand community priorities and culture (vii) Speak frankly and agree to disagree (viii) Include representation in the project from both those affected directly by the research and the community as a whole (ix) Evaluate the partnership frequently to elicit partners' feelings

(iii) Establish lines of communication	(i) Take time at the beginning to get to know one another and keep frequent contact with intended users (ii) Spend time in the community (e.g., attend significant community events) (iii) Jointly develop a written research agreement clearly spelling out roles and responsibilities of all partners (iv) Follow through on the agreement and any other promises (v) Hire community members as project staff

(iv) Form a community-led board	(i) Include wide representation from key community organizations where implemented (ii) Jointly develop operating norms including decision-making, conflict resolution, and meeting facilitation (iii) Adopt consensus decision-making (iv) Hold monthly meetings, rotate meeting locations if possible, and circulate draft agendas and meeting minutes (v) Include intended users in the management structure by hiring a respected community leader for their project's primary leadership role to facilitate community connections, share perspectives, and provide project oversight

(v) Group facilitation techniques; an iterative process when deciding upon research goals and grounded research question(s)	(i) Engage the project's advisory committee in a series of discussions with the community to incorporate local knowledge (ii) Establish working relationships early (iii) Consider having the community apply as principal applicant for grants

(vi) Build community capacity	(i) Utilize and develop community resources and support networks when conducting research (ii) Train community members as cofacilitators of research activities (iii) Involve the community in needs assessment and planning processes

(vii) Outline community involvement in research agreements	(i) The community can be involved in all phases of research (ii) Ensure active involvement of community members in all study tasks (e.g., reviewing all study documents to ensure they are in an understandable language) (iii) Solicit suggestions from community partners through focus groups or meetings (e.g., on data collection approaches) (iv) Hire and train lay community members or utilize an advisory board as field coordinators, interviewers, data collectors, intervention staff, and analysts (e.g., identification of variables, selection of measures, and questionnaire development)

(viii) Community training in research	(i) Provide training to community about health issues (ii) Use training sessions to get community perspective on these issues (iii) Have community members critique preexisting research instruments as a way of learning about developing questionnaires and for researchers to learn about the community's perspective (iv) Teach community public health and research skills (v) Conduct community workshops on research methods (vi) Use focus groups to engage community members in discussions about research in their community

(ix) Engage in early community interactions while developing the project	(i) Conduct in-depth interviews with community members and other key informants (ii) Go on “wind-shield” tours driving around the community (iii) Involve community in developing context-specific models (iv) Make use of qualitative data (v) Use theoretical, convenience, and open sampling

(x) Advisory committee	(i) Set up a subcommittee of the advisory committee to review all partnership evaluation results and make recommendations to the overall advisory committee (ii) Advisory committee can facilitate data analysis and interpret results (iii) Present and discuss results with community partners to facilitate interpretation (iv) Researchers and community members can analyze data independently and present their interpretations (v) Engage in open, interactive analysis with community partners (vi) Adopt a research agreement at the beginning outlining community involvement in results interpretation

(xi) Action planning	(i) Establish action groups of community partners to develop intervention strategies and plan policy initiatives (ii) Work with community members in deciding upon policy initiatives and action plans (iii) Instrumental use of research results to lobby government (iv) Work with community partners to develop community resources based upon study results (v) Hold meetings with community partners to discuss other nonstudy-related, important issues

(xii) Interpretation, data ownership, and dissemination	(i) Community partners can communicate their own interpretation of study data along with researcher study publications (ii) Adopt a no veto rule, meaning that neither researchers nor community partners can block a publication with results (iii) Spell out this process in a written researcher agreement before it arises (iv) Researchers can be guardians of the data during the project, but transfer data control to community after the project ends (v) Community obligation is to allow researchers the right to on-going data analysis (vi) Develop dissemination strategy outlining community involvement (vii) Include nonacademic partners as coauthors/copresenters on manuscripts/abstracts (viii) Disseminate results through local organizations, newspapers, media, and community-based practitioners (ix) Jointly publish a community newsletter with results included (x) Make use of local cultural mechanisms, such as street theatre (xi) Circulate a summary report to community members and/or have feedback/discussion sessions (xii) Organize debriefing sessions with a luncheon or gala celebration (xiii) Discuss publication drafts with the community before submission
